# A Quasi-Exclusive European Ancestry in the Senepol Tropical Cattle Breed Highlights the Importance of the *slick* Locus in Tropical Adaptation

**DOI:** 10.1371/journal.pone.0036133

**Published:** 2012-05-09

**Authors:** Laurence Flori, Mary Isabel Gonzatti, Sophie Thevenon, Isabelle Chantal, Joar Pinto, David Berthier, Pedro M. Aso, Mathieu Gautier

**Affiliations:** 1 INRA, UMR 1313 GABI, F-78350 Jouy-en-Josas, France; 2 CIRAD, UMR INTERTRYP, Montpellier, France; 3 Departamento de Biología Celular, Universidad Simón Bolívar, Caracas, Venezuela; 4 INRA, UMR CBGP, Montferrier-sur-Lez, France; University of Florence, Italy

## Abstract

**Background:**

The Senepol cattle breed (SEN) was created in the early XX^th^ century from a presumed cross between a European (EUT) breed (Red Poll) and a West African taurine (AFT) breed (N’Dama). Well adapted to tropical conditions, it is also believed trypanotolerant according to its putative AFT ancestry. However, such origins needed to be verified to define relevant husbandry practices and the genetic background underlying such adaptation needed to be characterized.

**Methodology/Principal Findings:**

We genotyped 153 SEN individuals on 47,365 SNPs and combined the resulting data with those available on 18 other populations representative of EUT, AFT and Zebu (ZEB) cattle. We found on average 89% EUT, 10.4% ZEB and 0.6% AFT ancestries in the SEN genome. We further looked for footprints of recent selection using standard tests based on the extent of haplotype homozygosity. We underlined i) three footprints on chromosome (BTA) 01, two of which are within or close to the *polled* locus underlying the absence of horns and ii) one footprint on BTA20 within the *slick* hair coat locus, involved in thermotolerance. Annotation of these regions allowed us to propose three candidate genes to explain the observed signals (TIAM1, GRIK1 and RAI14).

**Conclusions/Significance:**

Our results do not support the accepted concept about the AFT origin of SEN breed. Initial AFT ancestry (if any) might have been counter-selected in early generations due to breeding objectives oriented in particular toward meat production and hornless phenotype. Therefore, SEN animals are likely susceptible to African trypanosomes which questions the importation of SEN within the West African tsetse belt, as promoted by some breeding societies. Besides, our results revealed that SEN breed is predominantly a EUT breed well adapted to tropical conditions and confirmed the importance in thermotolerance of the *slick* locus.

## Introduction

Over the past decades, population genetic approaches associated with archeological evidence have provided better insights into the origin of modern livestock breeds including domestication processes, migration routes and their relationships [1]–[5]. The recent release of complete genome sequences and the development of high density SNP genotyping assays (e.g. [6]) in most livestock species have greatly increased available genomic information to refine characterization of breed origins and to efficiently detect footprints of selection in animal genomes. As a result, assembling large genetic datasets from livestock breeds, it is now possible to evaluate the accuracy of the reports on the origin of breeds which are widespread in the scientific literature. For instance, several publications referred to the Italian Piemontese cattle breed as a hybrid between the extinct aurochs and Indo-Pakistani Zebu. This myth was only recently refuted by genetic fingerprinting which showed that Italian Piemontese (as might have been expected) is a mixture of several European taurine (EUT) breeds [7]. Less fanciful, among the tropical cattle, the Kuri cattle breed has long been reported as an African taurine (AFT) breed in particular because these animals are humpless [8], characterized by a submetacentric Y chromosome from a taurine origin and devoid of Zebu allele on the Y chromosome [9]. However, nuclear markers such as microsatellites and SNPs showed that Kuri is actually a hybrid between AFT and Zebu (ZEB) breeds [10]–[15]. Apart from being of historical interest, such clarification of beliefs could be essential in the management and diffusion of some cattle breeds.

In addition, high density SNP dataset has highlighted chromosomal regions and genes targeted by artificial selection in dairy and/or beef cattle and by natural selection in West African cattle [14], [16]–[20]. Recently, by combining genetic structure analysis, examination of extended haplotype homozygosities (EHH,[21]–[23]) and detection of local excess or deficiency of a given ancestry relative to the average genome admixture level [24], we characterized the genetic origins of an admixed New World Creole cattle breed and detected footprints of selection in the genome.

Such a global approach is applied hereby on the Senepol cattle breed (SEN), a completely polled breed originating from the Virgin Islands, now widespread in several tropical regions. The SEN breed is particularly well adapted to tropical conditions, SEN animals being able for instance to maintain a normal body temperature during heat stress and appearing as heat tolerant as Brahman Zebu (BRA) cattle [25]–[27]. It is hypothesized that their sleek and shiny hair coat is responsible for or participates in their thermotolerance.

According to the St. Croix (US Virgin Island) breed society, SEN was created by Henry Nelthropp’s family at the beginning of 1900s on the St. Croix Island [28]. Nelthropp’s declared objective was to develop a hornless, early maturing breed, with gentle disposition, that combined the adaptation to tropical conditions of AFT cattle with meat production abilities of EUT cattle. He thus chose to cross supposed N’Dama (NDA) cows originating from Senegal with bulls belonging to the Red Poll cattle breed bought in Trinidad island and originating from Great Britain [29]. Since 1977, SEN have been spread in tropical areas around the world, in particular to some states of the US mainland, Australia and several countries of Latin America. The SEN Cattle Breeders Association (SCBA) recognized in 1999 over 500 breeders and more than 14,000 SEN records [30].

As an important consequence, many technical documents about SEN breed report that SEN individuals are considered to have inherited from their presumed NDA ancestry trypanotolerance characteristics, i.e. the ability displayed by West AFT breeds such as NDA to “survive, reproduce and remain productive under trypanosomiasis risk without trypanocidal drug” [31], [32]. SEN has thus been proposed by some breeding societies as a good candidate to improve production performances of local western African cattle living in areas infested by tsetse flies, the biological vectors of *Trypanosoma* (Associação Brasileira dos Criadores de Bovinos Senepol, http://senepol.org.br). To our knowledge, trypanotolerance has never been rigorously assessed in SEN, although failure to verify it could have dramatic consequences both for local breeders and for local breed diversity if importation of SEN animals were carried out towards West Africa. Moreover, the official version of the SEN origins, which represents the only argument to suggest a possible trypanotolerance of the SEN breed, has been disputed [33], [34]. De Alba early questioned the veracity of pure NDA importation in St. Croix and also noticed that supposed NDA animals harbored phenotypic characteristics of ZEB cattle [33]. He finally suggested that SEN animals could be derived from Creole cattle imported from the island of Viquez in St. Croix before the creation of the SEN breed. Although the official version of origin is routinely repeated by breed societies, the true SEN origin remains uncertain, but needs to be clarified before the breed is widely disseminated into tsetse infested areas.

Our study was primarily aimed at providing a detailed assessment of the genetic origins of the SEN using high density SNP data. To that purpose, a total of 153 SEN cattle were genotyped on the Illumina Bovine SNP50 chip and the resulting data were combined with those already available on 18 other breeds [6], [14]–[16]. In order to better characterize the origin of the adaptation to tropical conditions of the SEN breed, we further examined footprints of selection within this population using standard Extended Haplotype Homozygosity (EHH) based tests (e.g. [24]).

## Results

### The Genetic Characterization of SEN Cattle Breed Excludes AFT Ancestry

In order to characterize the SEN genetic history, we first combined the SNP data obtained on the 147 SEN animals (from the 153 genotyped SEN animals, see Materials and Methods) with those available on 18 worldwide cattle populations [14]–[16] corresponding to six European taurine (EUT), four AFT, four ZEB and four admixed breeds (one AFTxEUT, two AFTxZEB and one EUTxZEB), respectively. The combined data set consists of 623 individuals genotyped for 47,365 SNPs (see Materials and Methods).

The neighbor joining (NJ) tree based on allele sharing distance (ASD) separated individuals according to their population of origin (Figure S1). Breeds can also be grouped according to the three main populations (EUT, AFT and ZEB). Admixed breeds (OUL, KUR, BOR and SGT) branched at intermediate positions between their populations of origin. SEN animals from different origins (different Venezuelan states, St. Croix Island and USA) appear as a homogeneous group and branched at the same position than the SGT (EUT×ZEB).

**Figure 1:**
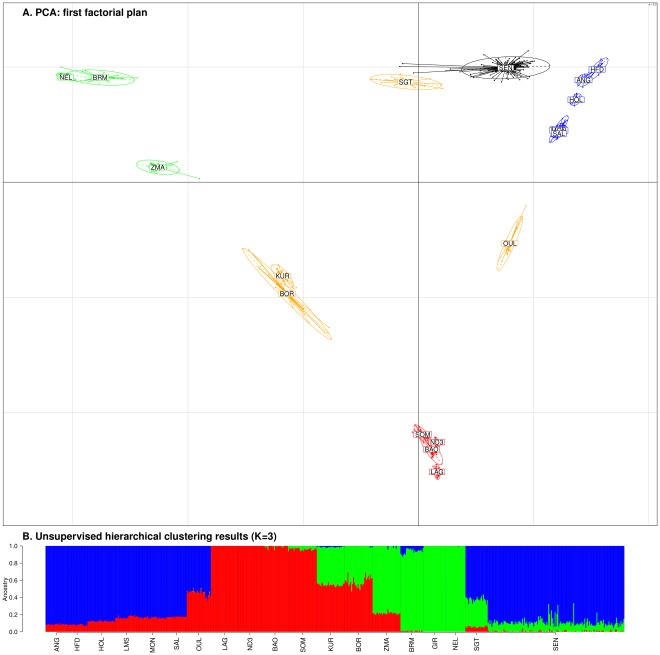
First factorial plan resulting from the PCA and unsupervised hierarchical clustering results. A. PCA was performed on 629 animals and 47365 SNPs. The first factorial plan is composed by PC1 and PC2 with corresponding eigenvalues equal to 7.91 and 6.22%. Ellipses characterize the dispersion of each population around its center of gravity. SEN individuals are plotted in black and individuals from the EUT, AFT, ZEB and hybrid (BOR, KUR, OUL and SGT) populations are plotted in blue, red, green and orange respectively. B. An unsupervised hierarchical clustering based on 629 individuals genotyped for 47365 SNPs is presented with an inferred number of clusters K = 3. For each individual, the proportion of each cluster (y) which were interpreted as representative of EUT, AFT and ZEB ancestries are plotted in blue, red and green, respectively.

An individual Principal Component Analysis (PCA) was then carried out using all available SNP information (Figure 1A). The first and the second components (PC1 and PC2) explained 7.91 and 6.22% of the variation, respectively. Focusing on this first factorial plan, we obtained the previously described triangle-like 2-Dimensional global organization of cattle genetic diversity [14], [15]. This triangle is shaped by EUT (in blue), AFT (in red) and ZEB (in green) at the three apexes. Hybrid breeds (in orange) such as SGT lay at their intermediated position in agreement with the NJ tree. Unexpectedly, SEN (in black) is positioned between SGT (EUTxZEB) and EUT on the side of the triangle limited by ZEB and EUT, at the opposite position from AFT. The SEN position suggests an influence of EUT and ZEB ancestries, with a greater EUT ancestry. Interestingly, PC2 also discriminates EUT populations according to a North/South gradient and SEN appeared closer on this axis to ANG and HFD (northern Europe breeds) than MON and SAL (southern Europe breeds).Using a model-based unsupervised hierarchical clustering of the individuals considering different K numbers of predefined clusters (Figures 1B and S2), we quantified the different SEN ancestry proportions. Results obtained with K = 3 were in agreement with PCA ones (Figure 1.B). The three clusters in dark blue (K1), red (K2) and green (K3) could be broadly interpreted as EUT, AFT and ZEB ancestry, respectively. SEN individuals had on average 89% (ranging from 66 and to 95%), 10.4% (ranging from 4 and to 33%) and 0.6% (ranging from 0 and to 4.1%) of EUT, ZEB and AFT ancestries, respectively. Hence, AFT ancestry in SEN individuals was even lower than that observed in individuals belonging to Northern-Europe breeds (Table S2). Increasing the number of clusters (K = 4) led to a similar overall picture for non-SEN individuals and the additional cluster (in green) isolated SEN individuals. Such a clustering of SEN individuals into a single group when K = 4 is essentially a consequence of overrepresentation of SEN individuals (n = 147) compared to other breeds (Table S1; Figure S2). Indeed, when a new analysis was performed, replacing the 147 initial SEN individuals with a sample of 30 randomly chosen SEN individuals (Figure S3), this trend was not observed. Note that the analysis on the reduced data sets essentially confirmed our previous observation for K = 3 and K = 4 and SEN individuals displayed a high percentage of Northern-Europe breed ancestry for K = 4.

Focusing now on the different SEN population origins, we observed i) a low level of differentiation among populations from the four different Venezuelan states represented (*F_ST_* <0.021), and ii) no inbreeding within the whole SEN population (|*F_IS_*|<0.01) (Table 1). This was consistent with the recent introduction of SEN in Venezuela and exchanges of animals. It justified in turn considering SEN populations as one single population.

**Table 1:** Heterozygosity (HZ), inbreeding coefficient (F_IS_) for each breed and differentiation (F_ST_) of SEN vs other breeds.

Origin	Breed	HZ	*F_IS_*	*F_ST_*
AFT	BAO	0.2080	−0.0123	0.2168
	LAG	0.1822	0.0193	0.2479
	NDA	0.2053	0.0085	0.2191
	SOM	0.2232	0.0500	0.2000
EUT	ANG	0.2998	0.0212	0.1428
	HFD	0.3077	0.0274	0.1558
	HOL	0.3050	0.0003	0.1385
	LMS	0.3026	0.0002	0.1113
	MON	0.2805	−0.0356	0.1417
	SAL	0.2808	0.0198	0.1363
HYB	BOR	0.2596	0.0042	0.1612
	KUR	0.2575	0.0058	0.1632
	OUL	0.2703	−0.0342	0.1431
	SEN	0.2912	0.0058	
	SGT	0.3039	−0.0028	0.1123
ZEB	BRM	0.1939	0.0289	0.2530
	GIR	0.1596	0.0093	0.2870
	NEL	0.1571	−0.0171	0.2869
	ZMA	0.1958	0.0174	0.2453

As expected, regarding the individual-based analyses (v Figures 1, S1 and S2), pairwise *F_ST_* between SEN and EUT breeds were lower than those between SEN and ZEB or AFT breeds (Table 1) and close to *F_ST_* between EUT breeds (Table S3). As shown in Table 1, SNP average heterozygosity for the SEN breed (0.29) was within the range observed in EUT breeds (from 0.28 to 0.30) and above those observed in AFT (from 0.18 to 0.22) or ZEB (from 0.15 to 0.19) populations.

### Identification of Footprints of Selection

**Figure 2:**
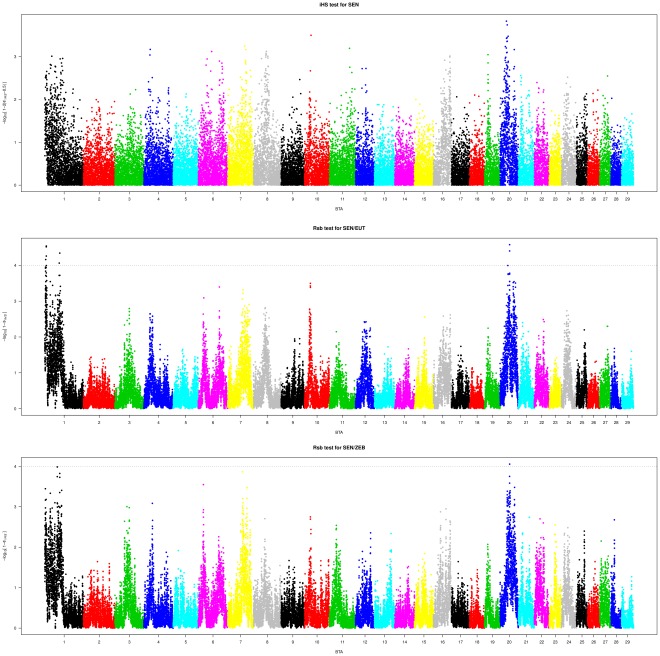
Plots over the genome of the SEN iHS (a) and ZEB/SEN (b) and EUT/CGU (c) Rsb scores for each SNP.

Following Gautier & Naves (2011) [24], we i) computed *iHS* scores [23] for each SNPs over the whole genome using the 294 SEN phased haplotypes [35] for each bovine autosome and ii) calculated *Rsb* statistics [22] to compare the local extent of haplotype homozygoties between SEN and its different ancestries (EUT and ZEB). For each autosome, haplotypes representative of EUT and ZEB ancestry, respectively, were pooled. Figure 2 represents the plots of the three different scores (*iHS* for SEN and *Rsb* for both EUT/SEN and ZEB/SEN comparisons).

**Figure 3:**
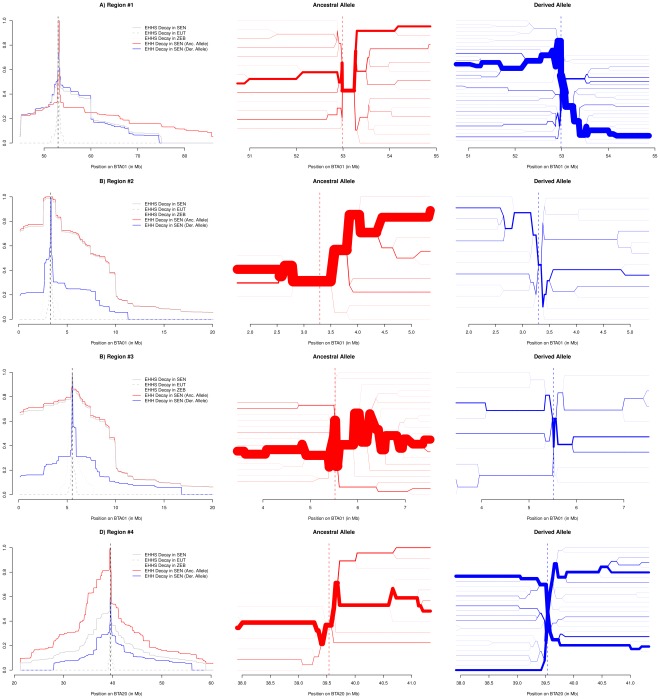
Details of the haplotype structure within the four significant footprints of selection identified. For each of the four regions, the decays of EHH (SEN ancestral and derived allele) and EHHS (for SEN, EUT and ZEB populations) from the core SNP located under the peak position are plotted in the leftmost panel. Haplotype bifurcation diagrams for both the ancestral (in red in the center panel) and derived allele (in blue in the rightmost panel) are also represented.

Table 2 summarizes characteristics of the four chromosomal regions displaying significant *iHS* and/or significant *Rsb* scores, three of which (#1, #2 and #3) map to BTA01 and one (#4) to BTA20. According to our somewhat stringent criteria, region #4 was significant for the three tests performed (*iHS*, *Rsb_EUT/SEN_* and *Rsb_ZEB/SEN_*), region #1 was significant for two tests (*iHS* and *Rsb_ZEB/SEN_*) and both regions #2 and #3 were found only significant with the *Rsb_EUT/SEN_* statistic. In order to illustrate the haplotype structure around the ancestral and derived alleles for each of the four SNPs at the peak position, we drew i) the decay of site-specific EHH or EHHS (weighted average of both EHH used for the computation of *Rsb*) within SEN, EUT and ZEB populations, ii) the EHH decay from the core SNP at both alleles within the SEN population and iii) haplotype bifurcation diagrams for both ancestral and derived allele in each region under selection within the SEN population (Figure 3). For Region#1, haplotypes containing the derived core SNP allele harbored a clear long-range Linkage Disequilibrium (LD) *i.e.* a thick branch in both directions from the core SNP. This suggests that the underlying causal variant is associated to this allele. Conversely, for regions #2 and #3, the underlying selected variant might be associated to the ancestral allele at the corresponding peak core SNP. For region #4, the signal is less clear since haplotype diversity is reduced at both the ancestral and derived alleles suggesting several favorable variants associated to various haplotype background might be under segregation (Figure 3). Following the UCSC UMD3 assembly, candidate genes could be identified at or close to the peaks for regions #2 (TIAM1: T-cell Lymphoma Invasion and Metastasis 1), # 3 (GRIK1: Glutamate Receptor, Ionotropic, Kainate 1) and #4 (RAI14: Retinoic Acid Induced 14). No candidate gene could be found for region #1.

**Table 2:** Chromosomal regions under selection.

Region	BTA	Position(Mb)	Peak position(Mb)	*iHS_SEN_*	*Rsb_EUT/SEN_*	*Rsb_ZEB/SEN_*	Nb of significant SNPs	Gene closest to the maximum
#1	1	52.6–53.6	52.9	3.984	NS	3.984	2	No gene found
#2	1	2.4–3.4	3.2	NS	4.161	NS	6	TIAM1
#3	1	4.7–5.8	5.5	NS	4.538	NS	8	GRIK1
#4	20	38.6–39.6	39.5	4.055	4.576	4.055	3–6	RAI14

## Discussion

### Revisiting the Origin of SEN Cattle Breed: Consequences for Breeding Strategies

Our study provides a first fine-scale genetic characterization of SEN through a comparison with other breeds representative of major breed groups of cattle distributed world-wide. The SEN individuals are representative of the whole population since they were sampled in different regions of Venezuela, and included some animals brought directly from the USA and the St. Croix Island, where the breed originated. No clear sub-structure was observed among the SEN individuals, suggesting an homogeneity of the population. In addition, SEN individuals displayed on average 89% of EUT ancestry meaning that SEN is predominantly a EUT breed. The origin of the EUT ancestry from North European breeds thus remains in agreement with historical and SEN breed association reports indicating that Red Poll individuals were initially used to create SEN. Although limited to 10.4% on average in SEN individuals, a ZEB ancestry was unexpectedly observed. More strikingly, given the very recent creation of the SEN breed (beginning of the XX^th^ century), our results clearly challenge the official historical version since no significant AFT ancestry was found in the SEN population. In order to explain such discrepancies, two hypotheses might be proposed. First, NDA breeding individuals were actually not used to create the SEN breed. As mentioned above, some authors raised doubts about a possible NDA ancestry [33], [34]. Hence, De Alba indicated that individuals participating in the breed creation in St.Croix had ZEB phenotypic characteristics suggesting Creole cattle (present in the St. Croix Island before the SEN breed creation) contributed to the SEN breed. Alternatively, AFT genome might not have been retained in the first individuals because AFT background had been counter-selected. We might for instance speculate that descendants from EUTxNDA crosses were not chosen as reproducers because of poor zootechnical characteristics or because they did not fit breeding objectives, one of them being hornless. Indeed, the inheritance of horns is mainly controlled by two locus: i) the *polled* locus located on BTA01 in *Bos Taurus* and ii) the *African horn* locus in *Bos taurus* and especially *Bos indicus*, which has an epistatic effect on the *polled* locus [36]. Based on the genetic determinism of horn development, NDA (horned) x RedPoll (completely polled) crosses result in polled F1 females and horned F1 males probably not retained as reproducers, leading to a counter-selection of individual carrying AFT ancestry.

Importantly, the absence of AFT ancestry makes it unlikely that SEN animals have inherited trypanotolerance trait from their ancestries although such ability represents the main argument of some breeder associations for the diffusion of SEN to West-African countries. Indeed, both EUT and ZEB cattle are recognized as trypanosusceptible. Similarly, SEN might not have acquired trypanotolerance during their recent history since Caribbean islands such as St. Croix are *Trypanosoma* free. Moreover, both *Trypanosoma congolense (T.congolense)* which is responsible for severe outcome of the disease in African livestock and its obligate vector (the tsetse fly) are absent in South America. SEN cattle are already present in South Africa, Botswana, Namibia and Zimbawe and some other African countries are planning to import SEN animals because they are more productive under tropical conditions than local breeds in a tsetse free context. It might be highly advisable to limit SEN importation to countries or areas outside of the tsetse belt.

### Detection and Characterization of Footprints of Selection within SEN

Such new insights into SEN origins raised another interesting question. Indeed, how can a breed with almost 90% of European cattle ancestry be well adapted to tropical conditions? Due to the recent origin of the SEN breed, it is tempting to speculate that such adaptation was inherited from the ZEB ancestry. ZEB, that are widespread in African, Asian and South America tropical areas, are indeed recognized for their thermotolerance, their ability to survive during food shortage and their resistance to several tick-borne diseases [37], [38].

In such a case, SEN represents an attractive model to identify those genomic regions from ZEB origin involved in its adaptation to tropical conditions. Alternatively, some favorable alleles might have segregated in EUT ancestry of the SEN breed in which theyhave subsequently been selected. To unravel loci potentially involved in adaptation of SEN to tropical conditions, we then searched for footprints of selection using previously described EHH-related tests (based on *iHS* and *Rsb* statistics). *iHS* based tests allow to detect loci carrying favorable variants subjected to strong recent or ongoing selection and display maximal power when they have not yet reached fixation [23]. Conversely, *Rsb* based tests show higher power to detect selective sweeps that have resulted in near or complete fixation of the favorable variant in one of the compared populations [22]. Four footprints of selection were then identified using *iHS* and *Rsb* statistics, three located on BTA01 (#1, #2 and #3) and one located on BTA20 (#4).

Interestingly, this latter region (#4) is within the previously described *slick* hair locus responsible for the very short, sleek hair coat [27], [39]. The slick phenotype was shown to be controlled by a single dominant unknown gene [27] located on a 4.4 cM region on BTA20 [39]. The resulting particular hair coat plays an important role in thermotolerance since hair coat thickness and hair weight per unit surface, are involved in adaptability to warm climate by limiting evaporative heat [40]. More precisely, Olson and colleagues showed that vaginal temperature, skin temperature and respiration rate of animals classified as slick was lower than normal-haired animals in ¾ Holstein ¼ Senepol crossbred cattle during acute heat stress [41]. This difference is partly due to an increase sweating rate [41], but could also result from an effective reflexion of solar radiations and an increase of heat loss via convection and conduction [42]. In addition, Olson and colleagues observed that the slick-haired dairy cows in Venezuela have an increased milk yield compared to normal haired animals [27]. Hence the *slick* locus appears as a strong candidate to explain the observed footprint of selection underlining thereby its importance in SEN adaptation to tropical conditions. Under this assumption, our study greatly refines the mapping interval of the underlying gene and allows us to propose a relevant positional and functional candidate gene. Indeed, the peak of the signal is located within the Retinoic Acid induced 14 gene (RAI14 or NORPEG), a developmentally regulated gene induced by all trans retinoic acid [43]. Retinoic acids are able to modulate several biological functions and are particularly involved in the control of hair follicule morphogenesis and cycling. Indeed, the prototypic retinoid all-trans retinoic acid is able to impair hair shaft elongation *in vitro* and can induce hair loss through premature hair follicule regression [44]. Topical treatment of human patients suffering from baldness, with retinoid all-trans retinoic acid in association with minoxidil enhances the hair follicule anagen-prolonging effects of the latter [45].

Among regions located on BTA01, no candidate genes could be found for region #1. However and interestingly, regions #2 and #3 mapped within (or close to in the case of region #3) the *polled* locus [46]. The polled phenotype corresponding to the absence of horns is caused by a single dominant mutation which has been mapped on BTA01 in several independent studies but has not yet been identified [36], [46]–[48]. Focusing on cattle breeds artificially selected for dairy production, other authors reported also a footprint of selection located in the *polled* locus defined in Drogemüller et al. and Wunderlich et al. studies [46], [48], [49]. To date, the most refined *polled* critical interval published extends from microsatellite BM6438 and RP42-218J17_MS1 on BTA01 [48] and is contained in a 2.5 Mb contig spanning the interval between SLC5A3 and SOD1 [46]. Assuming that the *polled* allele is responsible for the observed signals, our study allows us to propose two candidate genes lying under these peaks. TIAM1 encoding the T-cell lymphoma invasion and metastasis protein and GRIK1, encoding a ionotropic glutamate receptor, which are located in regions #2 and #3, respectively. TIAM1 modulates the activity of Rho-like proteins and connects extracellular signals to cytoskeletal activities [50]. This protein is crucial for the integrity of adherens junctions and cell matrix interactions [51]. The Glutamate receptor family, which comprises GRIK1, is involved in the mediation of excitatory synaptic transmission and plays a key role in cognitive function. Glutamate signaling is also involved in modifications of differentiation and osteoclasts and osteoblasts activities in bone [52]. Recently, Goto and colleagues showed that two ionotropic glutamate receptor genes (GRIN2C and GRIN3A) are the most divergent glutamate receptor genes between humans and chimpanzees [53]. GRIN3A showed also significant dN/dS acceleration in the human branch based on the analysis of six mammalian species [54]. A cluster of keratoproteins (KRTAP) is located between regions #2 and #3. In our previous study in West African cattle breeds, we also highlighted a region under selection near the *polled* region, with a peak near the KRTAP8-1 gene, located 0.5 Mb from TIAM1 and 1.38 Mb from GRIK1 [14].

For regions #2 and #3, examination of EHH decay and haplotype bifurcation diagrams suggested that the ancestral allele at the corresponding peak SNPs is associated to the underlying selected variant. For region #4, both ancestral and derived allele seem associated to an underlying selected variant. Due to the high EUT ancestry, ancestral alleles are thus more likely to be of EUT origin (see Materials and Methods). However, it remains difficult based on such observations to infer the origin of the selected variants. Nevertheless, a suggestive excess of local EUT ancestry [24] was observed in the footprints of selection observed in regions #2, #3 and #4 (data not shown). For regions #2 and #3, such observations are thus in agreement with a EUT origin of the hornless phenotype. Interestingly, for region #4 located within the *slick* hair locus (BTA20), the underlying favorable variant might be associated to several haplotype backgrounds from EUT ancestry. Discovered in SEN, the *slick* allele has also been described in other Creole breeds such as the Carora [27], Romosinuano (which is also polled) and Criollo Limonero which all have supposedly an important EUT ancestry. Fine scale genetic characterization of the SEN cattle breed allowed us to revisit the SEN breed origin. Since no AFT ancestry could be found in this population, the SEN breed might be viewed as a EUT breed well adapted to tropical conditions making it a relevant biological model to study adaptation to tropical climates. In addition, such results may help in the definition of management and breeding practices, namely to avoid importation of this breed to countries within the tsetse belt. Finally, we identified several footprints of selection presumably driven by artificial (selection toward the hornless phenotype) and natural (heat) pressures.

## Materials and Methods

### Ethics Statement

No ethics statement was required for the collection of DNA samples. DNA was extracted either from commercial AI bull semen straws or from blood samples obtained from different veterinary practitioners visiting farms with the permission of the owners.

### Selection of Animals, Blood Sampling and Genotyping

A total of 153 SEN individuals from Venezuela were included in our genetic study. In Venezuela, the first importation of SEN cattle was done in 1988, in the form of 250 frozen embryos from American Senepol Limited (Harrogate, Tenessee), which were implanted in Zebuin and Holstein breeds between 1988 and 1992. In 1991, the first bull was imported and 30,000 doses of its semen were sold. In May 1993 a total of 200 animals were imported from Saint Croix to seven different farms in Venezuela. Up to 2011, 540 animals have been imported from St. Croix or USA, for a total of 1,774 fullblood and purebred Senepol animals and 4,974 crossbred animals that are participating in the upgrade program in Venezuela. The Venezuelan Association of Senepol Cattle Breeders (ASOSENEPOL) was created in 1998 and has 25 active members spread in the territory and 13 veterinarians that classify the cattle in the whole country. They have a collaborative program with the international Senepol Cattle Breeders Association.

These 153 SEN animals (Table S1) belong to four Venezuelan states (39, 20, 36 and 58 animals collected in Guarico, Anzoategui, Bolivar and Monagas, respectively) and 10 different farms (two, one, three and four breeding units located in Guarico, Anzoategui, Bolivar and Monagas, respectively). Among them, 134, 13 and five are from Venezuelan, St. Croix and USA origin. Special care was taken to choose pure SEN animals belonging to the herdbook and to limit animal relationships based on available pedigree information.

Blood samples were collected in EDTA Vacutainer tubes and DNA was extracted using the Wizard® Genomic DNA Purification Kit (Promega, France). DNA quantity and quality were evaluated on Nanodrop and on agarose gels (1% stained with SYBR safe, Invitrogen).

The 153 animals (Table S1) were genotyped on the second version of the Illumina BovineSNP50 chip (v2) at INRA Labogena plateform (Jouy-en-Josas, France) using standard procedures (http://www.illumina.com) [6]. Six SEN individuals were further removed from subsequent analyses because they were genotyped for less than 95% of SNPs. We completed the SEN genotyping data with SNPs genotypes already obtained using the first version of the Illumina BovineSNP50 chip (v1) for 21 to 30 individuals belonging to18 other breeds (Table S1) representative of i) EUT: SAL, LMS, HOL, MON, ANG, HFD, ii) AFT : BAO, SOM, LAG, ND3, iii) ZEB: NEL, GIR, BRM, ZMA and iv) four crossbreed populations : OUL (EUTxAFT), BOR (AFTxZEB), KUR (AFTxZEB), SGT (EUTxZEB) [14], [15]. The complete data set finally consists of genotypes for 623 individuals including 147 SEN individuals. Both versions of the Illumina BovineSNP50 chip have 52340 SNPs in common. Among these SNPs, we discarded from the analyses SNPs genotyped for less than 75% of the individuals per breed.

Following Gautier et al (2010) an exact test for Hardy-Weinberg Equilibrium (HWE) was subsequently carried out within each breed separately on the remaining SNPs [55] and q-values were estimated for each SNP using the R package qvalue (http://cran.r-project.org/web/packages/qvalue/index.html) [56]. SNP with a q-value below 0.1 in at least one population were discarded leading to a total of 47,365 SNPs in the final data set.

### Analyses of Population Structure

F-statistics *F_IT_*, *F_ST_* and *F_IS_*, and the diversity estimation for each locus and population both within and among individuals within a population were estimated using the program GENEPOP 4.0 [57].The within breed *F_IS_* was derived from the average of these two quantities over all the SNPs.

A neighbor-joining tree [58] based on Allele Sharing Distance (ASD) was computed with APE R package [59]. For a given pair of individuals i and j, ASD was defined as 1-x_ij_ where x_ij_ represents the proportion of alleles alike in state averaged over all genotyped SNPs.

Principal Component Analysis (PCA) and unsupervised hierarchical clustering of individuals based on SNP genotyping data were performed using the smartpca software package and the program Admixture 1.04, respectively [60], [61]. PCA results were visualized using the R package ade4 [62], [63].

### Detection of Footprints of Selection

Haplotypes were estimated using fastphase 1.4 for SEN and each AFT, ZEB and EUT populations [35]. We further computed *iHS* and *Rsb* score [22], [23] using haplotype information. To compute *iHS*, the SEN ancestral allelic state was defined as the highest ancestral frequency estimated as *fa = w_EUT_ f_EUT_+w_ZEB_f_ZEB_*, were *w_EUT_* and *w_ZEB_* represent the average proportions of EUT and ZEB ancestries in the SEN genome and *f_EUT_* and *f_ZEB_* the allele frequency of EUT and ZEB clusters [24]. The different quantities were estimated using Admixture 1.04 [60] as described above. For convenience SNP *iHS* scores were further transformed into *p_iHS_* = −log[1–2|Φ(*iHS*)−0.5|] where Φ(x) represents the Gaussian cumulative function. Assuming *iHS* scores are normally distributed under neutrality (Figure S4), *p_iHS_* might thus be interpreted as *log_10_*(1/P) where P is the two-sided *p-value* associated to the neutral hypothesis (no selection). All the above analyses and plots (including bifurcation diagrams) were performed using the newly developed R package rehh [64].

*Rsb* was computed for two comparisons (EUT/SEN and ZEB/SEN) by standardizing the ratio of the corresponding ancestral cluster (EUT or ZEB) *iES* and the SEN *iES* [22]. SNP scores were transformed into *p_Rsb_* = -log[Φ(*Rsb*)]. As above, assuming *Rsb* are normally distributed (under neutrality), *p_Rsb_* might be interpreted as log_10_(1/P) where P is the one-sided *p-value* associated to the neutral hypothesis (no selection).

### Identification and Annotation of Candidate Regions

For each 1 Mb window over the genome (with a 0.5 Mb overlap), the candidate regions were identified by counting the number of SNP with *p_iHS_* >4 (P<0.0001) and similarly the number of SNP with *p_Rsb_* >4 for each of the two comparisons (EUT/SEN and ZEB/SEN). Regions containing at least two SNPs exceeding these thresholds for at least one test were considered as candidate. When several overlapping and contiguous windows were candidates, the chosen one contained the highest proportion of significant SNPs and also the peak. The candidate regions were then annotated using the UCSC Genome browser (http://genome.ucsc.edu) and the *Bos taurus* UMD 3.1/bosTau6 assembly. A gene was considered a candidate if the peak position was located <25 kb from its boundaries.

## Supporting Information

Figure S1 **Neighbor-Joining tree relating the 629 individuals.** Among the 623 individuals, 147 SEN from different origins and states and 476 animals from 18 other breeds were analysed. The tree was constructed using allele sharing distances averaged over 47365 SNPs. Edges are colored according to the individual breed of origin.
(TIF)

Figure S2 **Unsupervised hierarchical clustering results with different number of clusters of 623 individuals genotyped for 47365 SNPs.** The 623 animals, comprising 147 SEN individuals, were analyzed with an inferred number of clusters K = 2 (A), K = 3 (B), K = 4 (C), K = 5 (D) and K = 6 (E). For each individual, the proportion of each cluster (y) which were interpreted as representative of EUT, AFT and ZEB ancestries for K = 3.
(TIF)

Figure S3 **Unsupervised hierarchical clustering results with different number of clusters of 506 individuals genotyped for 47365 SNPs.** The 506 individuals, comprising 30 SEN individuals randomly chosen, were analyzed with an inferred number of clusters K = 2 (A), K = 3 (B) and K = 4 (C). For each individual, the proportion of each cluster (y) which were interpreted as representative of EUT, AFT and ZEB ancestries are plotted in blue, red and green, respectively.
(TIF)

Figure S4 **Empirical distribution of SEN, EUT and ZEB** ***iHS*** **scores.**
(EPS)

Table S1 **List of population symbols, names and number of individuals.**
(XLS)

Table S2 **Percentage of the corresponding AFT, ZEB and EUT ancestries for each breed.**
(XLS)

Table S3 **Differentiation score (** ***Fst*** **) for all pairs of breeds.**
(XLS)
