# Supplementary figures and images for: A Quasi-Exclusive European Ancestry in the Senepol Tropical Cattle Breed Highlights the Importance of the slick Locus in Tropical Adaptation
Source: PLoS One. 2012 May 9;7(5):e36133. doi: 10.1371/journal.pone.0036133 (PMC3366548; doi:10.1371/journal.pone.0036133)

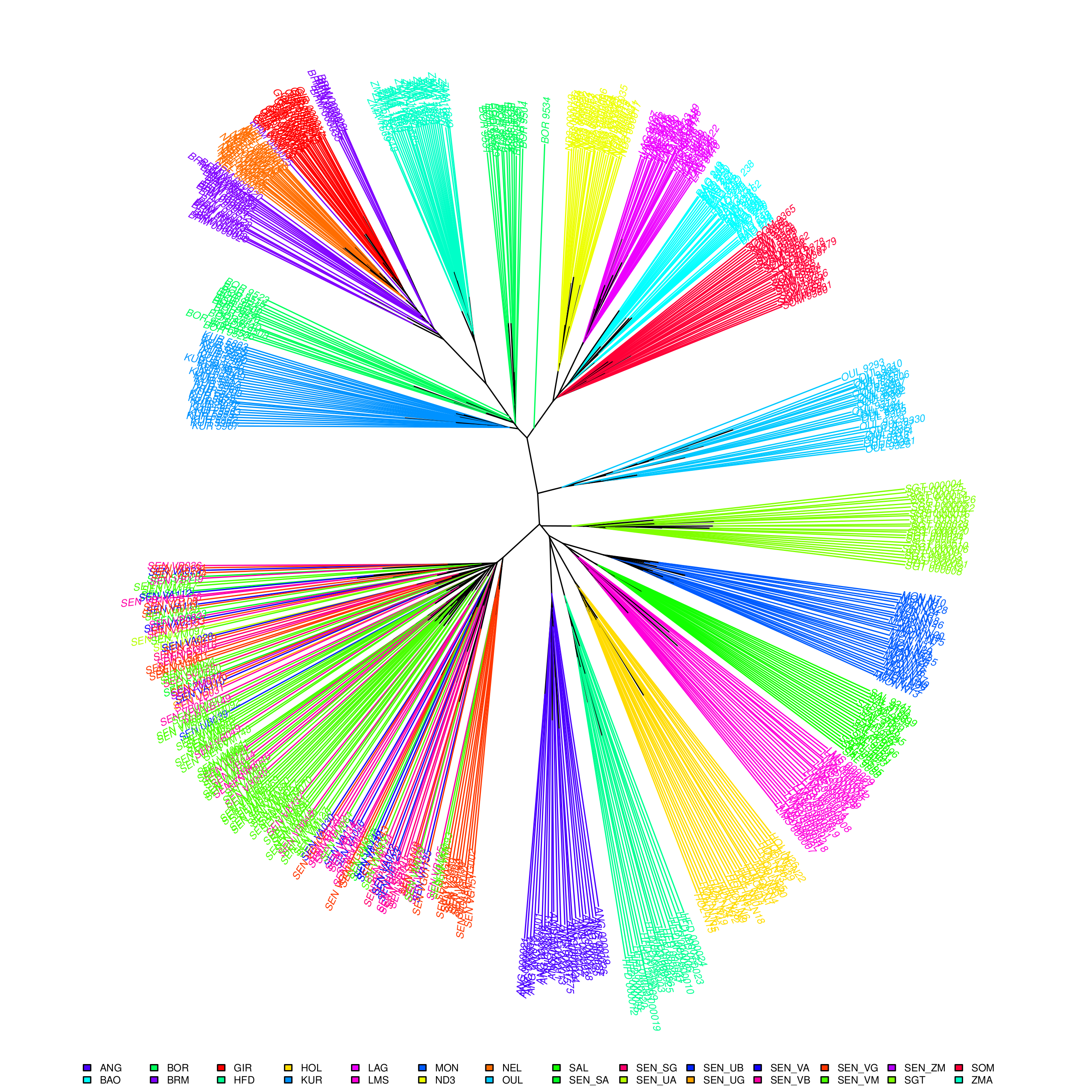

Supplement: Figure S1 — Neighbor-Joining tree relating the 629 individuals. Among the 623 individuals, 147 SEN from different origins and states and 476 animals from 18 other breeds were analysed. The tree was constructed using allele sharing distances averaged over 47365 SNPs. Edges are colored according to the individual breed of origin. (TIF) [file pone.0036133.s001.tif]

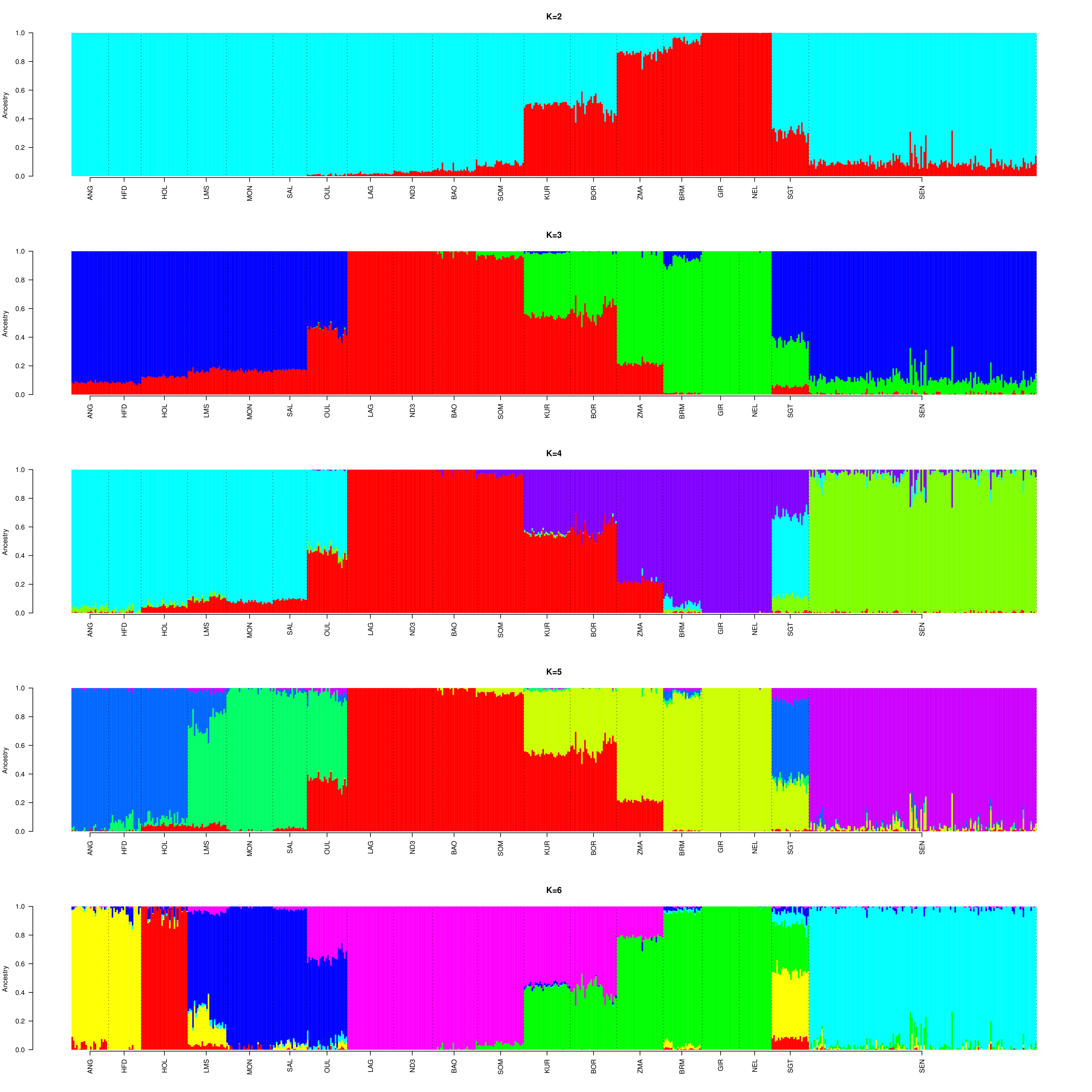

Supplement: Figure S2 — Unsupervised hierarchical clustering results with different number of clusters of 623 individuals genotyped for 47365 SNPs. The 623 animals, comprising 147 SEN individuals, were analyzed with an inferred number of clusters K = 2 (A), K = 3 (B), K = 4 (C), K = 5 (D) and K = 6 (E). For each individual, the proportion of each cluster (y) which were interpreted as representative of EUT, AFT and ZEB ancestries for K = 3. (TIF) [file pone.0036133.s002.tif]

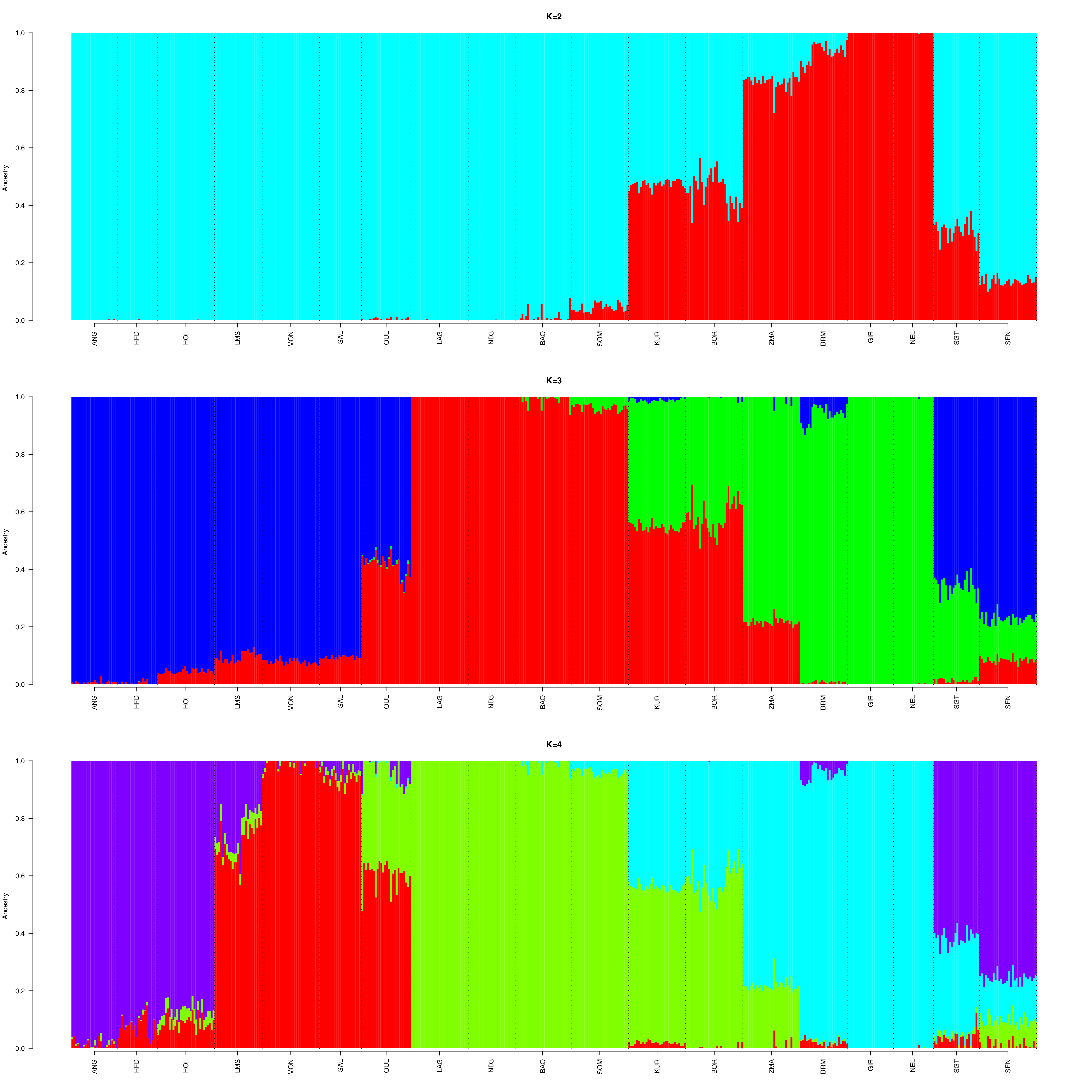

Supplement: Figure S3 — Unsupervised hierarchical clustering results with different number of clusters of 506 individuals genotyped for 47365 SNPs. The 506 individuals, comprising 30 SEN individuals randomly chosen, were analyzed with an inferred number of clusters K = 2 (A), K = 3 (B) and K = 4 (C). For each individual, the proportion of each cluster (y) which were interpreted as representative of EUT, AFT and ZEB ancestries are plotted in blue, red and green, respectively. (TIF) [file pone.0036133.s003.tif]
